# Supplementary material for: HIVprotI: an integrated web based platform for prediction and design of HIV proteins inhibitors
Source: J Cheminform. 2018 Mar 9;10:12. doi: 10.1186/s13321-018-0266-y (PMC5845081; doi:10.1186/s13321-018-0266-y)
Supplement: Supplementary file 2 — Additional file 2. Source code of HIVProtI web server. [file 13321_2018_266_MOESM2_ESM.zip › HIVprotI_Source-code/hivprotI/weka-3-6-6/doc/weka/attributeSelection/ASSearch.html]

ASSearch


---


|  |  |  |
| --- | --- | --- |
| **Overview** \| **Package** \| **Class** \| **Tree** \| **Deprecated** \| **Index** \| **Help** \| **Weka's home** | |  |
| **PREV CLASS**   **NEXT CLASS** | **FRAMES**    **NO FRAMES**     **All Classes** |  |
| SUMMARY: NESTED \| FIELD \| CONSTR \| METHOD | DETAIL: FIELD \| CONSTR \| METHOD |  |


---


## weka.attributeSelection Class ASSearch

```
java.lang.Object
  weka.attributeSelection.ASSearch
```

**All Implemented Interfaces:**: java.io.Serializable, RevisionHandler

**Direct Known Subclasses:**: BestFirst, ExhaustiveSearch, GeneticSearch, GreedyStepwise, LinearForwardSelection, RaceSearch, RandomSearch, Ranker, RankSearch, ScatterSearchV1, SubsetSizeForwardSelection

---

``` public abstract class ASSearch extends java.lang.Object implements java.io.Serializable, RevisionHandler ```

Abstract attribute selection search class.

**Version:**
:   $Revision: 1.12 $

**Author:**
:   Mark Hall (mhall@cs.waikato.ac.nz)

**See Also:**: Serialized Form

---

**Constructor Summary**

`ASSearch()`


| **Method Summary** | |
| --- | --- |
| `static ASSearch` | `forName(java.lang.String searchName, java.lang.String[] options)`             Creates a new instance of a search class given it's class name and (optional) arguments to pass to it's setOptions method. |
| `java.lang.String` | `getRevision()`             Returns the revision string. |
| `static ASSearch[]` | `makeCopies(ASSearch model, int num)`             Creates copies of the current search scheme. |
| `abstract  int[]` | `search(ASEvaluation ASEvaluator, Instances data)`             Searches the attribute subset/ranking space. |

**Methods inherited from class java.lang.Object**

`equals, getClass, hashCode, notify, notifyAll, toString, wait, wait, wait`

**Constructor Detail**

### ASSearch

```
public ASSearch()
```


**Method Detail**

### getRevision

```
public java.lang.String getRevision()
```

:   Returns the revision string.

    :   **Specified by:**: `getRevision` in interface `RevisionHandler`
    :   **Returns:**: the revision

---


### search

```
public abstract int[] search(ASEvaluation ASEvaluator,
                             Instances data)
                      throws java.lang.Exception
```

:   Searches the attribute subset/ranking space.

    :   **Parameters:**: `ASEvaluator` - the attribute evaluator to guide the search: `data` - the training instances. **Returns:**: an array (not necessarily ordered) of selected attribute indexes **Throws:**: `java.lang.Exception` - if the search can't be completed

---


### forName

```
public static ASSearch forName(java.lang.String searchName,
                               java.lang.String[] options)
                        throws java.lang.Exception
```

:   Creates a new instance of a search class given it's class name and
    (optional) arguments to pass to it's setOptions method. If the
    search method implements OptionHandler and the options parameter is
    non-null, the search method will have it's options set.

    :   **Parameters:**: `searchName` - the fully qualified class name of the search class: `options` - an array of options suitable for passing to setOptions. May be null. **Returns:**: the newly created search object, ready for use. **Throws:**: `java.lang.Exception` - if the search class name is invalid, or the options supplied are not acceptable to the search class.

---


### makeCopies

```
public static ASSearch[] makeCopies(ASSearch model,
                                    int num)
                             throws java.lang.Exception
```

:   Creates copies of the current search scheme. Note that this method
    now uses Serialization to perform a deep copy, so the search
    object must be fully Serializable. Any currently built model will
    now be copied as well.

    :   **Parameters:**: `model` - an example search scheme to copy: `num` - the number of search scheme copies to create. **Returns:**: an array of search schemes. **Throws:**: `java.lang.Exception` - if an error occurs


---


|  |  |  |
| --- | --- | --- |
| **Overview** \| **Package** \| **Class** \| **Tree** \| **Deprecated** \| **Index** \| **Help** \| **Weka's home** | |  |
| **PREV CLASS**   **NEXT CLASS** | **FRAMES**    **NO FRAMES**     **All Classes** |  |
| SUMMARY: NESTED \| FIELD \| CONSTR \| METHOD | DETAIL: FIELD \| CONSTR \| METHOD |  |


---
